# Isolation of a Defective Prion Mutant from Natural Scrapie

**DOI:** 10.1371/journal.ppat.1006016

**Published:** 2016-11-23

**Authors:** Ilaria Vanni, Sergio Migliore, Gian Mario Cosseddu, Michele Angelo Di Bari, Laura Pirisinu, Claudia D’Agostino, Geraldina Riccardi, Umberto Agrimi, Romolo Nonno

**Affiliations:** 1 Department of Veterinary Public Health and Food Safety, Istituto Superiore di Sanità, Rome, Italy; 2 Istituto Zooprofilattico Sperimentale of Sicily "A. Mirri", Palermo, Italy; 3 Istituto Zooprofilattico Sperimentale dell'Abruzzo e del Molise "G. Caporale", Teramo, Italy; Creighton University, UNITED STATES

## Abstract

It is widely known that prion strains can mutate in response to modification of the replication environment and we have recently reported that prion mutations can occur *in vitro* during amplification of vole-adapted prions by Protein Misfolding Cyclic Amplification on bank vole substrate (bvPMCA). Here we exploited the high efficiency of prion replication by bvPMCA to study the *in vitro* propagation of natural scrapie isolates. Although *in vitro* vole-adapted PrP^Sc^ conformers were usually similar to the sheep counterpart, we repeatedly isolated a PrP^Sc^ mutant exclusively when starting from extremely diluted seeds of a single sheep isolate. The mutant and faithful PrP^Sc^ conformers showed to be efficiently autocatalytic *in vitro* and were characterized by different PrP protease resistant cores, spanning aa ∼155–231 and ∼80–231 respectively, and by different conformational stabilities. The two conformers could thus be seen as different *bona fide* PrP^Sc^ types, putatively accounting for prion populations with different biological properties. Indeed, once inoculated in bank vole the faithful conformer was competent for *in vivo* replication while the mutant was unable to infect voles, *de facto* behaving like a defective prion mutant. Overall, our findings confirm that prions can adapt and evolve in the new replication environments and that the starting population size can affect their evolutionary landscape, at least *in vitro*. Furthermore, we report the first example of “authentic” defective prion mutant, composed of brain-derived PrP^C^ and originating from a natural scrapie isolate. Our results clearly indicate that the defective mutant lacks of some structural characteristics, that presumably involve the central region ∼90–155, critical for infectivity but not for *in vitro* replication. Finally, we propose a molecular mechanism able to account for the discordant *in vitro* and *in vivo* behavior, suggesting possible new paths for investigating the molecular bases of prion infectivity.

## Author Summary

Prions are unique infectious agents, consisting of PrP^Sc^, a self-propagating aggregated conformer of the host-encoded prion protein PrP^C^. Despite the absence of any nucleic acid information, prions exist as distinct strains that share the same amino acid sequence but differ in their conformation. Moreover, prions can mutate and are thus heterogeneous populations able to evolve and adapt to new replication environments. During *in vitro* amplification of sheep scrapie, we found that a prion mutant could be obtained from one natural isolate. The prion mutant identified was characterized *in vivo* and *in vitro*, showing unusual biochemical and biological features: a smaller than usual C-terminal proteinase resistant core of PrP^Sc^, which spans aa ∼155–231, and the inability to propagate *in vivo* despite an efficient autocatalytic replication *in vitro*. With such a signature, we denoted the mutant as a “defective” prion mutant. We thus postulate a new hypothesis for the discrepancy between the *in vitro* and *in vivo* behavior of the defective mutant and suggest that the central PrP^Sc^ domain ∼90–160 might have a key role in prion replication. This work provides important new insights into the mechanism underpinning prion replication and has numerous implications for understanding the molecular requirements indispensable for prion infectivity.

## Introduction

Transmissible spongiform encephalopathies (TSEs) are progressive and fatal neurodegenerative disorders affecting animals and humans, with the most common forms being scrapie of sheep and goats, bovine spongiform encephalopathy (BSE) of cattle, chronic wasting disease (CWD) of cervids, and Creutzfeldt-Jakob disease (CJD) in humans. TSEs are caused by the misfolding of the host-encoded prion protein monomers (PrP^C^) into autocatalytic self-replicating aggregates (PrP^Sc^). The protein-only hypothesis postulates that the agent responsible for these pathologies, the prion, is exclusively composed of PrP^Sc^ [1]. Prion replication can be modelled as a template-based mechanism, where PrP^C^ misfolds into PrP^Sc^ and acquires the ability to recruit other PrP^C^ molecules and to trigger their misfolding, through an autocatalytic template-based mechanism [1]. This model is supported by protein misfolding cyclic amplification (PMCA) [2], a technique that mimics *in vitro* the PrP^C^-to-PrP^Sc^ autocatalytic conversion, leading to the generation of a huge amount of infectious prions in healthy brain homogenates seeded with minute amounts of PrP^Sc^ and subjected to multiple cycles of sonication and incubation [3].

Although devoid of a nucleic acid genome, prions exist as strains. Increasing evidence supports the view that prion strains are encoded by distinct PrP^Sc^ conformers [4–6]. Strain features are usually kept unchanged on serial passages in the same host; however, seminal studies reported that prion strains can mutate when crossing the species barrier or even on sub-passage in the same host species [7].

The phenomenon of prion mutation has been replicated in *ex vivo* [8, 9] and *in vitro* studies, mimicking inter- [10, 11] and intra-species [12, 13] transmission, thus highlighting the dynamic nature of prions in response to modifications in the replication environment. Furthermore, we have recently observed the emergence of mutant PrP^Sc^ conformers during *in vitro* amplifications even in the absence of PrP sequence mismatches, PrP^C^ modifications, RNA-depletion or treatments with drugs [14], supporting the idea of the quasispecies nature of prions [6, 15].

Bank voles have been shown to be highly sensitive to sheep scrapie [16, 17]. Accordingly, bank vole brain homogenates were an efficient substrate for the *in vitro* amplification of scrapie by PMCA [18]. This prompted us to investigate the sensitivity of bank vole PMCA to scrapie by limiting dilution experiments [17]. During these studies, we found that the propagation at limiting dilutions of a scrapie isolate resulted in strain mutation. Here we report the emergence, the isolation, the biochemical features and the biological characterization of the prion mutant identified. Interestingly, the prion mutant was characterized by an extremely C-terminal PK resistant core (PrP^res^) and arose exclusively from extremely diluted scrapie seeds. Moreover, it could be denoted as a defective prion mutant, since it was efficiently autocatalytic *in vitro*, but unable to propagate *in vivo*. These findings indicate that the defective prion mutant, although derived from a natural scrapie isolate, lacks of some essential structural characteristics for being infectious. We propose a molecular mechanism able to account for this discordant *in vitro* and *in vivo* behaviour, so as to highlight new paths for investigating the molecular underpinnings of prion infectivity.

## Results

### *In vitro* emergence of different PrP^Sc^ conformers from natural scrapie

**Fig 1:**
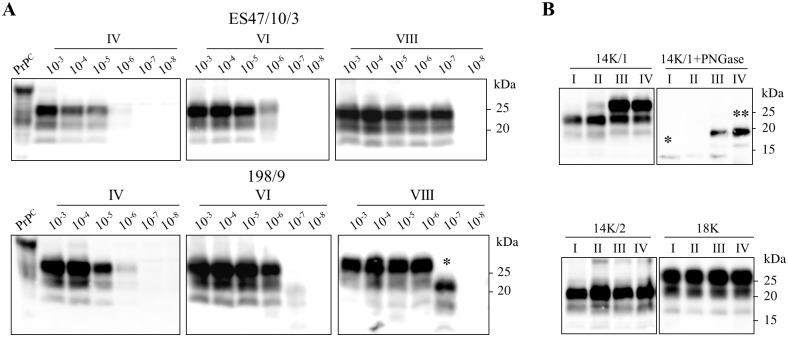
Identification of 14K from a natural scrapie sample. **A**) Serial 10-fold dilutions of 2 Italian scrapie samples (ES47/10/3 and 198/9) were used as seeds in serial PMCA reactions using vole brain homogenate substrate. Products from rounds 4°, 6° and 8° (indicated in roman numbers) were digested with PK and analyzed by Western blot with antibody SAF84. After 8 PMCA rounds both samples were positive up to dilution 10^−7^. An atypical PrP^Sc^ with smaller PrP^res^ (indicated by the asterisk) emerged after the sixth round only from the last detectable dilution of sample 198/9, and was propagated until the end of the experiment. **B**) Three *in vitro* selected prion populations (18K, 14K/1 and 14K/2, as indicated on the top of each blot) were serially propagated for 4 successive PMCA rounds (represented in roman numbers). After each round, aliquots of the PMCA products were digested with PK and analyzed by Western blot with antibody SAF84. For 14K/1, PK-digested PrP^res^ is also shown after enzymatic removal of N-linked oligosaccarides, which allows to better appreciate the co-presence and evolution of 14K and 18K PrP^res^ (indicated by a single and a double asterisk respectively) during the experiment.

To test the sensitivity of detection of serial bank vole PMCA (bvPMCA) on natural scrapie isolates, serial ten-fold dilutions of 3 sheep scrapie isolates (198/9, ES47/10/2 and ES47/10/3) were subjected to 10 rounds of PMCA (Fig 1A). The sensitivity observed with the whole set of inocula resembled that reported by Chianini et al. [17], showing a limit of detection of 10^−6^/10^−7^ after 8 bvPMCA rounds (Fig 1A). Sixteen unseeded samples were used as negative controls and remained negative up to the 10^th^ round, showing that neither cross-contamination nor spontaneous appearance of PrP^Sc^ occurred under these experimental conditions, as previously reported [18]. Moreover, with the only exception of dilution 10^−7^ of 198/9 (see below), the electrophoretic pattern of the PK-resistant core of the amplified PrP^Sc^ was similar to that in the original inocula, as previously observed with other scrapie isolates [17]. We will refer to this pattern as 18K, according to the apparent MW of the unglycosylated PrP^res^.

Unexpectedly, a different PrP^Sc^ type was obtained from the dilution 10^−7^ of 198/9 after 6 PMCA rounds. This atypical PrP^Sc^ type, characterized by a PrP^res^ of ~14 kDa (molecular weight of unglycosylated band) and thus named 14K, emerged only from the last detectable dilution of sample 198/9 and preserved its biochemical features for two further bvPMCA rounds (Fig 1A). The experiment was repeated two more times, starting from the same three scrapie seeds. Again, this atypical PrP^Sc^ type emerged only from the last detectable dilutions of 198/9, respectively 10^−5^ after 7 rounds of PMCA and 10^−7^ after 4 rounds. In order to exclude that the atypical PrP^Sc^ could have arisen by the exposure of bank vole substrates to diluted sheep brain seeds, serial ten-fold dilutions, from 10^−5^ to 10^−7^, of brain homogenates from 3 healthy sheep were subjected to 7 rounds of PMCA. None of these negative seeds induced the amplification of 18K or 14K PrP^Sc^ (S1 Fig).

### *In vitro* serial amplification and isolation of PMCA-derived 18K and 14K prion populations

We selected two of the 14K samples obtained from the isolate 198/9 derived from the dilution 10^−7^ after 8 bvPMCA rounds (named 14K/1) and the dilution 10^−5^ after 11 rounds (named 14K/2), as well as an 18K sample derived from the dilution 10^−5^ after 6 bvPMCA rounds from the same scrapie isolate.

The three samples were subjected to serial bvPMCA in order to produce sufficient amount of material for further analyses, and they all confirmed to be autocatalytic *in vitro*. However, while 18K and 14K/2 preserved faithfully their biochemical characteristics, 14K/1 gradually shifted to an 18K profile (Fig 1B), being composed of both 14K and 18K PrP^res^ after 2 bvPMCA rounds and of only 18K PrP^res^ after 2 other rounds, as revealed by deglycosylation studies (Fig 1B).

**Fig 2:**
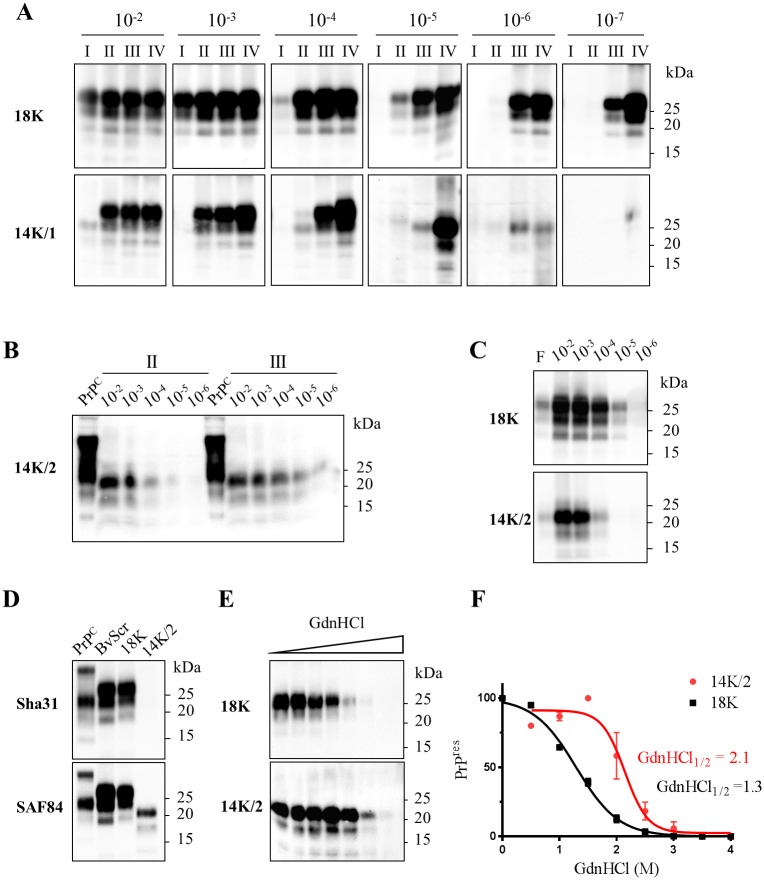
Isolation and biochemical characterization of 18K and 14K PrP^Sc^. **A**) Serial ten-fold dilutions of 18K and 14K/1 PrP^res^ (10^−2^ to 10^−7^) were subjected to 4 successive PMCA rounds (expressed in roman numbers) and PrP^res^ was analyzed by Western blot after each round. While 18K PrP^res^ alone was detectable in the whole set of dilutions seeded with 18K (upper side of the panel), both the PrP^res^ types emerged from sample 14K/1, i.e. 18K PrP^res^ from the low dilutions of the curve and 14K PrP^res^ from the high ones, indicating the mixed nature of the sample. **B**) Serial ten-fold dilutions of sample 14K/2 were propagated for 3 PMCA rounds. PrP^res^ obtained after the 2^nd^ and 3^rd^ rounds faithfully preserved the 14K signature. **C**) In order to compare their *in vitro* autocatalytic efficiency, serial ten-fold dilutions of 18K and 14K/2 were subjected to a single PMCA round, PK digested, analyzed by Western blot and the PrP^res^ detected with antibody SAF84. For each curve the inoculum not subjected to PMCA was added (first lane, F). **D**) Epitope mapping of PrP^res^ from vole-adapted scrapie (BvScr), 18K and 14K/2. PrP^C^ from a negative vole brain homogenate not treated with PK was also included as control. Membranes probed with Sha31 and SAF84 antibodies are shown, as indicated on each blot. **E**) Representative Western blot of CSA experiments with 18K and 14K/2. Aliquots of the samples were treated with increasing concentrations of GdnHCl (from 0 to 4 M) and residual PrP^res^ was analyzed by Western blot. **F**) Dose-response curves were obtained by plotting the mean fraction of PrP^res^ detected as a function of GdnHCl concentration and best fitted using a four parameter logistic equation. [GdnHCl]_1/2_ values are indicated in the graph.

The reappearance of 18K from 14K/1 could be explained by the presence of minimal amounts of 18K in the sample, which outcompeted 14K during successive PMCA rounds. To test this hypothesis, we reasoned that we could get rid of any remaining 18K in 14K/1 by a dilution approach. Indeed, serial 10-fold dilutions of 14K/1 subjected to 4 serial rounds of bvPMCA showed that 18K emerged only from the lowest dilutions, while 14K was preserved from dilution 10^−5^ to 10^−7^ (Fig 2A). Moreover, 14K further preserved its signature for other 4 rounds, confirming that 14K/1 was composed of a mixture of a huge quantity of 14K and minute amounts of 18K, that could be selectively removed by dilution.

As our aim was to obtain a 14K as pure as possible, we focused our attention on sample 14K/2, which did not shift to 18K in previous experiments. Five more rounds of bvPMCA did not revealed any 18K profile from sample 14K/2. To get rid of any possible 18K we then subjected 14K/2 to the same dilution approach used for 14K/1 (Fig 2B). The two last positive dilutions of the 3^rd^ round (10^−5^ and 10^−6^) were thus pooled, diluted 1:100 in vole brain homogenate, split in 28 tubes and subjected to 2 rounds of PMCA, in order to produce a large amount of a pure 14K/2 population. Thus, after a total of 21 rounds of PMCA and a cumulative dilution factor of 10^−31^, a “cloned” 14K was obtained from the scrapie isolate 198/9.

We then tested the efficiency of *in vitro* replication of 18K and 14K/2, subjecting a serial ten-fold dilution curve of the two samples to bvPMCA amplification. 18K dilutions up to 10^−6^ were positive after a single round, while the 10^−5^ dilution of 14K/2 was still negative (Fig 2C). The finding that 18K was ~100 times more efficient than 14K/2 further corroborates our previous hypothesis that in 14K/1 a minimal 18K component outcompeted 14K during serial PMCA.

### Biochemical characterization of PMCA-derived 18K and 14K PrP^Sc^ types

Deglycosylation experiments showed that both 18K and 14K/2 PK-resistant cores were composed of C-terminal, fully glycosylated PrP fragments. Epitope mapping of 18K and 14K/2 showed that 18K was similar to vole-adapted scrapie and it was recognized by the antibodies with epitopes from SAF32 (directed to the octarepeat region of PrP) to the C-terminus (Fig 2D and S2 Fig) indicating to be composed of the expected ~80–231 PrP^res^ fragment, similar to the PrP^res^ previously observed in sheep and voles with classical scrapie [19, 20]. In contrast, only the C-terminal antibody SAF84 (aa 163–169) was able to recognize 14K/2 (Fig 2D and S2 Fig). The absence of the Sha31 epitope (aa 145–152) in the PK-resistant core of 14K/2 indicated that PrP^Sc^ was cleaved by proteinase K between the aa 152 and 163, thus spanning aa ~155–231. Deglycosylation of 18K and 14K/2 confirmed that both PrP^res^ types were composed of single variably glycosylated C-terminal PrP fragments (S2 Fig).

We then assessed the PrP^Sc^ conformational stability of 18K and 14K/2 by denaturation with increasing GdnHCl concentrations. Again, the two bvPMCA-derived PrP^Sc^ types had distinct features, as 14K/2 showed a conformational stability higher than 18K, with GdnHCl values of 2,1 and 1,3 M respectively (Fig 2E and 2F). Overall, based on the different PK-cleavage site and conformational stability observed, these results imply that PrP^Sc^ types with PrP^res^ cores of 18K and 14K are self-replicating *bona fide* PrP^Sc^ aggregates with distinct conformations.

### Vole bioassay: 14K as a defective prion mutant

We tested the infectivity and the strain properties of bvPMCA-derived PrP^Sc^ 18K, 14K/1 and 14K/2 by bioassay in vole, in comparison with the original scrapie isolate 198/9.

**Fig 3:**
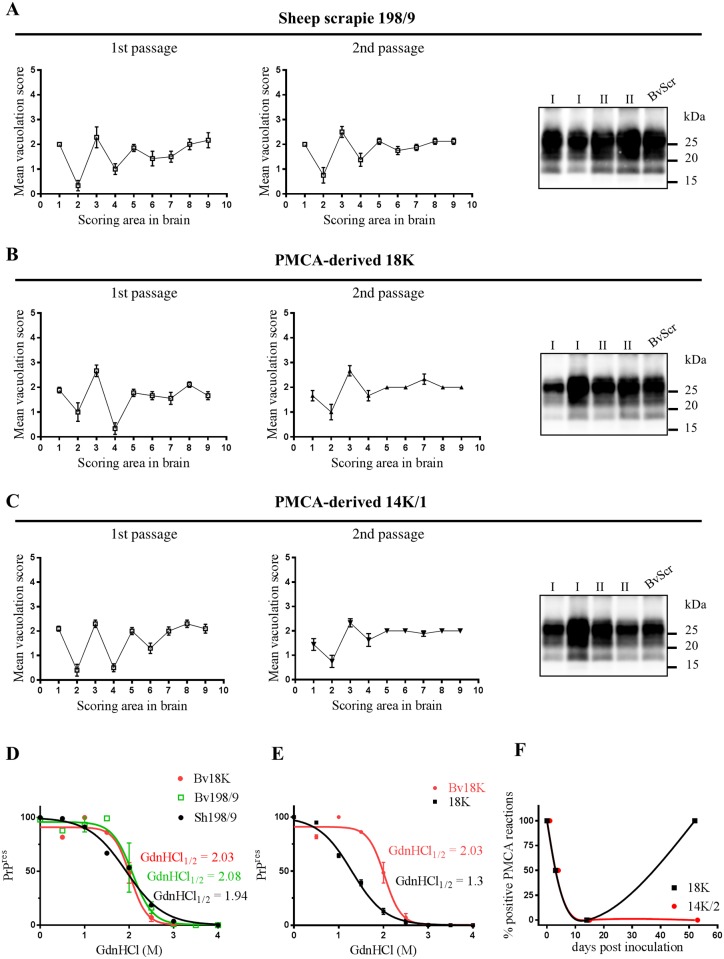
Vole bioassay. **A**, **B** and **C** show the neuropathological and PrP^res^ phenotypes observed in voles after primary transmission (left panels, and indicated as I, roman number, in the blot on the right) and second passage (central panels and indicated as II, roman numbers, in the blot) of sheep 198/9 (**A**), PMCA-derived 18K (**B**) and PMCA-derived 14K/1 (**C**). Brain-scoring areas in lesion profiles are: medulla (1), cerebellum (2), superior colliculus (3), hypothalamus (4), thalamus (5), hippocampus (6), septum (7), retrosplenial and adjacent motor cortex (8), cingulate and adjacent motor cortex (9). For each blot a vole adapted scrapie was added (BvScr, last lane). PrP^res^ was detected by antibody SAF84. **D**) Graph depicting the denaturation profiles obtained by CSA from PrP^Sc^ in sheep 198/9 (Sh198/9) or from voles infected with 18K (Bv18K) and vole-adapted 198/9 (Bv198/9). **E**) Graph depicting the comparison of denaturation profiles of 18K before (18K) and after (Bv18K) transmission in voles. [GdnHCl]_1/2_ values are reported in the graph. **F**) Graph depicting the fate of 18K and 14K/2 after intracerebral inoculation in voles. Two groups of 8 voles were inoculated with 14K/2 or 18K and 2 voles for each group were sacrificed at different time points (0, 3, 14 and 52 dpi). Their brains were homogenized and used as seed for PMCA reactions in 3 independent experiments. The values on y axis represent the overall percentage of positive samples per time point after 3 PMCA rounds.

The scrapie isolate 198/9 transmitted to 100% of voles with a mean survival time of 167 days post infection (dpi). The survival time shortened to 94 dpi on sub-passage, indicating the existence of a transmission barrier for adaptation of sheep scrapie to voles (Table 1). The neuropathological phenotype (Fig 3A) was indistinguishable from that previously observed in other ARQ/ARQ scrapie isolates from Italy and UK [16], and all voles showed the expected 18K PrP^res^ profile with no evidence of shorter PrP^res^ fragments (Fig 3A). In previous studies we showed that PrP^Sc^ from several Italian classical scrapie isolates displayed a uniform conformational stability, with GdnHCl_1/2_ values of ~2 M [19, 20], which was preserved after transmission in voles [19, 21]. Accordingly, the conformational stabilities of 198/9 and vole-adapted 198/9 were similar, with GdnHCl_1/2_ of ~2 M (Fig 3D).

**Table 1:** Transmission of 198/9, 18K, 14K/1 and 14K/2 in bank voles.

	Primary Transmission		Second passage	
Inoculum	Mean survival time ± SD (days) †	Attack rate n/N° ǂ	Mean Survival Time ± SD (days)	Attack rate n/N° ǂ
**198/9**	167 ± 13	9/9	94 ± 3	8/8
**18K**	117 ± 9	9/9	92 ± 4	8/8
**14K/1**	154 ± 12	9/9	96 ± 5	9/9
**14K/2**	> 697	0/9	n.a.	n.a.

^†^ When voles do not show clinical signs, the range of survival time is reported.

^ǂ^ n, Number of mice that developed confirmed TSE disease;

N° number of inoculated mice.

BvPMCA-derived 18K was even more efficient than the original scrapie isolate, giving a shorter survival time in the first passage, 117 dpi, probably due to its *in vitro* adaptation. Indeed, on second passage the survival time showed only a minor shortening and converged to that observed for vole-adapted 198/9 (Table 1). The neuropathological phenotype matched that observed in voles infected with the scrapie isolate 198/9, and the 18K PrP^res^ profile was preserved upon *in vivo* propagation (Fig 3B). In contrast, the conformational stability of the 18K inoculum was not preserved *in vivo*, as vole-adapted 18K showed a rightward shift in the denaturation curve and a GdnHCl_1/2_ value of ~2 M (Fig 3E), thus fully converging with vole-adapted 198/9 (Fig 3D). These findings indicate that the same prion strain was isolated by both *in vitro* and *in vivo* adaptation of natural scrapie to voles, despite the divergent conformational stabilities of *in vitro* and *in vivo* derived PrP^Sc^ types.

BvPMCA-derived mutant 14K/1, which supposedly contained a mixture of 14K and 18K, efficiently transmitted in voles too, with a survival time of 154 dpi (Table 1). The survival time and the neuropathological phenotype at second passage were similar to those observed with 198/9 and 18K (Fig 3C). Furthermore, as previously observed *in vitro*, the PrP^res^ profile of 14K/1 shifted to 18K during *in vivo* replication (Fig 3C).

In contrast to all other samples, 14K/2 was unable to cause disease in voles, as none of the inoculated animals showed clinical signs up to 697 dpi (Table 1). All voles sacrificed for intercurrent disease or found dead were negative by neuropathological assessment and by WB for brain PrP^Sc^.

The inability of 14K/2 to infect voles was in sharp contrast with its efficient *in vitro* propagation. We thus determined the kinetics of *in vivo* PrP^Sc^ clearance and replication of intracerebrally inoculated 14K/2 and 18K. Two groups of 8 voles were inoculated with 14K/2 or 18K and 2 voles for each group were sacrificed at different time points (0, 3, 14 and 52 dpi); brains of voles were homogenized and used as seed for PMCA reactions (Fig 3F). Both 18K and 14K/2 were easily detectable at 0 dpi, barely detectable at 3 dpi and become undetectable at 14 dpi, indicating a similar trend of clearance for both PrP^Sc^ types. However, at 52 dpi 18K became strongly positive, indicating an active replication in vole brain, while 14K/2 remained undetectable. We further attempted to detect 14K/2 seeding activity at later time points, by using the brain of 9 voles inoculated with 14K/2 and sacrificed for intercurrent disease between 168 and 697 dpi, as well as the spleen of 2 voles sacrificed at 588 and 697 dpi. Again all tissues were negative when tested in a 3-rounds serial bvPMCA able to detect very low levels of PrP^Sc^. These findings imply that 14K/2, containing the “cloned” 14K PrP^Sc^, not only did not induce disease in voles, but was unable to replicate *in vivo* at levels sufficient to self-sustain in brain and spleen tissues.

## Discussion

In agreement with previous observations [17], our findings show that sheep PrP^Sc^ is able to efficiently propagate on vole PrP^C^ by PMCA, even when seeded at extremely high dilutions (up to 10^−7^), allowing to derive *in vitro* vole-adapted prion populations starting from a seemingly low number of replicative units. As expected, after several rounds of PMCA we usually recovered a faithful vole-adapted scrapie PrP^Sc^, characterized by a PrP^res^ of 18 kDa; however, from high dilutions of a single sheep isolate, 198/9, we repeatedly derived a PrP^Sc^ with a shorter PK-resistant core of 14 kDa. We selected and studied two prion populations, named 18K and 14K/2, both derived *in vitro* by serial bvPMCA, starting from highly diluted seeds of the same scrapie isolate. PrP^res^ from 18K and 14K/2 populations had different PK-cleavage site and different conformational stability, so that they could be seen as different *bona fide* PrP^Sc^ conformers, potentially encoding for different biological properties. Indeed, 18K was competent for *in vivo* replication and resulted in a pathological phenotype indistinguishable from vole-adapted 198/9 while, unexpectedly, 14K/2 was unable to infect voles. These results suggest that the *in vitro* derived prion population containing 18 kDa PrP^res^ aggregates encoded for the faithful scrapie strain, while that exclusively composed of 14K kDa PrP^res^ aggregates behaved like a defective mutant, being unable to replicate in live animals. It is of note, however, that even 18K did not preserve 100% fidelity compared to its *in vivo* counterpart, sheep and vole-adapted scrapie; indeed, the conformational stability of sheep and vole-adapted PrP^Sc^ was higher than that of 18K, which however reverted to the original phenotype upon *in vivo* propagation.

Several experimental approaches have been used to investigate the phenomenon of prion mutation. The derived observations gave rise to two different theories, not necessarily mutually exclusive: the “deformed templating model” by Ilia Baskakov and colleagues which postulates that the templating process is imperfect and changes in the replication environment play an active role in the *de novo* generation of new PrP^Sc^ variants [22–24], and the “cloud” model postulated by Charles Weissman and John Collinge [6, 9, 15, 25, 26] which proposes that even cloned strains consist of a cloud of different PrP^Sc^ conformers and that changes in the replication environment give selective advantage to the “fittest” among pre-existing variants. The first hypothesis would suggest that the 14K mutant could have been somehow induced by the *in vitro* replication environment of PMCA, while the latter hypothesis implies that the original scrapie isolate was already composed of a cloud of PrP^Sc^ conformers, then subjected to evolutionary constraints in the new *in vitro* replication environment. Whatever the molecular mechanism that underlies prions mutation and evolution, it looks undeniable that prions can adapt and evolve when propagated under particular selection regimes. Our findings are in line with previous observations and corroborate the notion that an agent that apparently lacks any nucleic acid information is still able to adapt in response to changes in the replication environment. Moreover, the fact that the mutant PrP^Sc^ emerged exclusively and repeatedly from the highest dilution of the isolate 198/9 further validates the hypothesis that the starting population size may have a profound impact on the evolutionary landscape of scrapie, at least *in vitro* [14]. These observations can be supportive of the “cloud” hypothesis, i.e. that prion populations behave as quasispecies. Indeed, in viral quasispecies, successive bottleneck passages mediate the surfacing of minority components present or generated in the mutant spectra of the original population. This is because mutant spectra are not mere distributions of neutral mutants, but they can hide components that in isolation would display dissimilar biological properties [27]. In this scenario, the starting population size may affect the evolutionary outcome of a given prion population under a new selective environment, such as a new host or any other *in vivo* or *in vitro* evolutionary constraint, which has important implications for understanding the inter- and intra-host biological variability of prions.

In previous studies, *ex vivo* and *in vitro* selected prion mutants had the tendency to revert to the parental strain upon *in vivo* propagation [9, 28]. In the present study, one mutant population, 14K/1, reverted to the parental strain when propagated *in vivo*, while the other, 14K/2, did not and was unable to propagate at all. Overall, our and previous studies suggest that most *in vitro* selected prion mutants may have a lower fitness than their parental strains for *in vivo* propagation. As far as mutants still contain minor populations of the parental strains, these would then easily reemerge *in vivo*. Our results strongly support this hypothesis, by showing i) that minor populations of 18K were actually present in the mutant 14K/1, which reverted to the parental strain *in vivo*, and ii) that 14K populations propagated *in vitro* under conditions able to get rid of any 18K (Fig 2) were indeed defective for *in vivo* propagation.

It seems difficult to reconcile the findings that 14K PrP^Sc^ was suitably competent for autocatalytic self-propagation *in vitro*, but lacked any infectivity *in vivo*. *In vitro* studies with recombinant PrP have shown the spontaneous emergence of autocatalytic but noninfectious recombinant PrP fibrils characterized by ~160–231 C-terminal PK-resistant core [29–31]. Compelling evidences suggested that PrP fibrils with more extended C-terminal PK-resistant core is required for fully competent *in vivo* replication [28, 29].

The present findings show that a similar phenomenon could be observed with natural scrapie and suggest that autocatalytic but noninfectious PrP^Sc^ is not merely an artificial product of recombinant PrP, but can derived from infectious PrP^Sc^ under experimental conditions able to replicate faithful and infectious prions.

Usually, full infectious PrP^Sc^ contains a PK-resistant core spanning residues ~ 90–231, which characterizes most known natural TSEs. We have recently found that PrP^Sc^ with PK-resistant core as short as that spanning residues ~ 100–145 [21] may still be highly infectious [32]. In contrast, all the above mentioned “defective” prions have in common a shorter than usual C-terminal PrP^res^, which do not include the central PrP domain. This PrP domain contains the polybasic lysine cluster (residues 101–110, vole PrP numbering), which has been suggested as a key structural modulator in the conversion of PrP^C^ to PrP^Sc^, either by binding to anionic cofactors that promote prion replication [31] or by mediating proper PrP^C^/PrP^Sc^ interaction [33]. Interestingly, PrP^Sc^ aggregates that lack the central polybasic domain showed to be autocatalytic *in vitro* but barely infectious *in vivo* [33].

**Fig 4:**
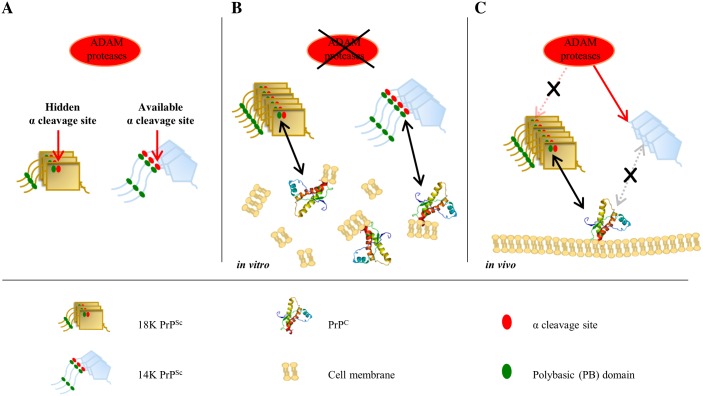
Hypothetical mechanisms underpinning the defective nature of 14K PrP^Sc^. The cartoon depicts the hypothetical interaction between physiological proteases responsible for α-cleavage, here supposed to belong to the family of ADAM proteases [35] and 18K or 14K PrP^Sc^ (**A**), and how it changes in the *in vitro* (**B**) and the *in vivo* (**C**) replication environments. Distinct symbols indicate PrP^C^ monomers, 18K or 14K PrP^Sc^ aggregates, as well as the α-cleavage site and the location of the polybasic domains in PrP^Sc^, as shown in the graphical legend below the cartoon. (**A**) In 18K PrP^Sc^ aggregates the physiological α-cleavage site (residues 110–111, vole PrP numbering) and the central polybasic domain (in green, aa ~ 101–110), are tightly packed in the PK-resistant core of PrP^Sc^. On the contrary, in mutant 14K PrP^Sc^ the α-cleavage site is available for hydrolysis. (**B**) During *in vitro* propagation by PMCA, the activity of ADAM proteases is purposely prevented by protease inhibitors, a factor which allows to keep full length PrP in solution. Under these conditions, both 18K and 14K PrP^Sc^ are full length and preserve intact polybasic domains, which would allow them to interact with PrP^C^ and replicate. (**C**) *In vivo*, 18K PrP^Sc^ is still protected from ADAM proteolysis as the cleavage site is buried within the PK-resistant core, and thus it is still fully competent for replication as it retains the N-terminus. In contrast, 14K PrP^Sc^ would be cleaved at 110–111 and would lose the central polybasic domain, supposed to be a key mediator of the interaction with partners indispensable for prion replication (PrP^C^ or cofactors).

The dominant PrP^C^ processing event, α-cleavage, occurs at the start of the hydrophobic core region, at approximately K110↓H111 (vole PrP numbering) [34, 35], producing the membrane anchored C-terminal C1 fragment and releasing the corresponding N-terminal N1 fragment, which contains the polybasic domain. This cleavage event is prevented in infectious PrP^Sc^, as the α-cleavage site is in the tightly packed PK-resistant core and is solvent excluded, but it is allowed in PrP^Sc^ with less extended ~ 155–231 PK-resistant core, in which residues 110–111 are solvent exposed and available to the endoproteolytic processing. Interestingly, noninfectious PrP^Sc^ with ~ 155–231 PK-resistant core have only been observed *in vitro* under conditions in which α-cleavage do not occur, i.e. in experiments involving purified preparations of recombinant PrP or in brain homogenates supplemented with protease inhibitors, that are routinely added in PMCA reactions to prevent PrP^C^ proteolysis. This observation prompted us to hypothesize that preserving PrP^Sc^ from α-cleavage could be indispensable for prion replication. In Fig 4 we describe a molecular mechanism based on this hypothesis, which is able to reconcile the contrasting *in vitro* and *in vivo* behavior of 14K PrP^res^. The model proposes that the short 100–110 polybasic peptide containing the lysine cluster plays a crucial role in prion replication and that the infectivity of 14K PrP^Sc^ was prevented by the removal of the polybasic domain through *in vivo* α-endoproteolysis, which is structurally inhibited in infectious 18K PrP^Sc^. Overall, taking into account the hypothesis of the quasispecies nature of prion strains, we might suppose that even defective PrP^Sc^ conformations such as 14K could be continuously generated and eliminated by the host endoproteolitic cleavage, i.e. the *in vivo* negative selection. Once the negative selection is abrogated, as occurs in PMCA experiments in which brain homogenates are supplemented with protease inhibitors, such innate continuous generation of PrP^Sc^ conformational variants might eventually result in the selective emergence of defective mutants.

In conclusion, several studies have been conducted to understand what PrP^Sc^ needs to be a prion; however, most, if not all, of these studies were built on recombinant PrP aggregates and, as such, have to deal with the community skepticism towards synthetic prions. To our knowledge, our findings provide the first “authentic” defective prion mutant, composed of brain-derived PrP^C^ and originating from a natural scrapie isolate. Our findings could provide new insights for dissecting the molecular mechanisms that differentiate autocatalytic PrP^Sc^ amplification from *in vivo* prion replication.

## Material and Methods

### Ethics statement

Bank voles carrying methionine at codon 109 were obtained from the breeding colony at the Istituto Superiore di Sanità (ISS). Experiments involving animals adhered to the guidelines contained in the Italian Legislative Decree 116/92, which transposed the European Directive 86/609/EEC on Laboratory Animal Protection, and then in the Legislative Decree 26/2014, which transposed the European Directive 2010/63/UE on Laboratory Animal Protection. The research protocol was performed under the supervision of the Service for Biotechnology and Animal Welfare of the ISS, and was approved by the Italian Ministry of Health (decree number 84/12.B).

### PMCA

Substrates were prepared according to the protocol previously reported [18]. Brain tissues from three ARQ/ARQ scrapie infected sheep (198/9, ES47/10/2 and ES47/10/3), from three ARQ/ARQ healthy sheep and from voles inoculated with 18K or 14K/2, or spleen tissues from voles inoculated with 14K/2, were homogenized in PBS (10% w/v) containing Complete Protease Inibitor Cocktail (Roche), divided into small aliquots and stored at -20°C. Serial ten-fold dilutions of the homogenates (a single tube per dilution from 10^−2^ to 10^−8^, where 10^−2^ means 1% w/v of infected brain homogenate) were prepared in PMCA substrate and were subjected to serial PMCA. PMCA was performed using the Misonix S3000 sonicator, following the procedures described in Cosseddu et al. [18]. The *in vitro* amplification was performed in a total volume of 50 μl for each dilution, and the sonication program consisted of 20 seconds sonication pulses every 30 minutes for 48 hours, at a constant temperature of 37°C. At the end of each round, 5 μl of each reaction mix were diluted 1:10 in fresh substrate for a new amplification round.

### Western blot analysis

PMCA reactions were added with an equal volume of Tris-HCl sarcosyl 4% and then digested with 100 μg/ml of proteinase K (Sigma-Aldrich) for diagnosis and 200 μg/ml for epitope mapping. Samples were shaken at 750 rpm for one hour at 55°C. The digestion was then stopped by adding 3 mM PMFS (Sigma). For epitope mapping, PK digested samples were added with an equal volume of isopropanol/butanol (1:1 v/v) and centrifuged at 20,000 g for 5 min. Supernatants were discarded and the pellets were suspended in denaturing sample buffer (NuPAGE LDS Sample Buffer, Invitrogen) and heated for 10 min at 90°C. Electrophoresis and Western blotting were performed as previously described [21]. The membranes were then analyzed with anti-PrP monoclonal antibody SAF84 (aa 167–173; 1,2 μg/ml), Sha31 (aa 146–152; 0,6 μg/ml), 9A2 (aa 99–101; 1 μg/ml), and SAF32 (octarepeat; 4,8 μg/ml). Following incubation with horseradish peroxidase-conjugated anti-mouse immunoglobulin (Pierce Biotechnology) at 1:20000, the PrP bands were detected by enhanced chemiluminescent substrate (SuperSignal Femto, Pierce) and VersaDoc imaging system (Bio-Rad). The chemiluminescence signal was quantified by QuantityOne software (Bio-Rad).

Deglycosylation was performed by adding 18 μl of 0.2 M sodium phosphate buffer (pH 7.4) containing 0.8% Nonidet P40 (Roche) and 2 μl (80 U/ml) di N-Glycosidase F (Roche) to 5 μl of PK-digested and denaturated samples and by incubating overnight at 37°C with gentle shaking. Samples were then analysed by Western blotting as described above.

### Conformational stability assay

To normalize the buffer condition of all the samples (*in vivo* and *in vitro* produced) subjected to CSA, brains from voles inoculated with 198/9 and 18K and from sheep 198/9 were homogenized in conversion buffer (PBS 1x, pH 7,4; 0,15 M NaCl; 1% Triton X with the Roche Complete Protease Inibitor Cocktail). The CSA was performed as described [19]. Briefly, brain homogenates and PMCA-derived products (both 6% w/v) were added with an equal volume of TrisHCl 100 mM (pH 7.4) containing sarkosyl 4% and incubated for 1h at 37°C with gentle shaking. Aliquots of 25 μl were added with 25 μl of GdnHCl to give a final concentration ranging from 0 to 4.0 M. After 1 h of incubation at 37°C all samples were diluted to a final concentration of 0.4 M GdnHCl and then PK digested (50 mg/ml PK final concentration) for 1 hour at 55°C and gentle shaking. The reaction was stopped with 3 mM PMSF (Sigma). Aliquots of samples were added with an equal volume of isopropanol/butanol (1 1 v/v) and centrifuged at 20000 g for 10 min. Pellets were re-suspended in NuPage LDS Sample Buffer (Invitrogen) and were analysed by Western Blotting as described above.

### Bioassay and neuropathological assessment

Brain tissues were homogenized at 10% (w/v) in phosphate buffered saline (PBS) and stored at -80°C. PMCA-derived inocula were diluted with PBS for a final concentration of 1% (w/v) and stored at -80°C. Groups of eight-week-old voles were inoculated intracerebrally with 20 μl of homogenate into the left cerebral hemisphere, under ketamine anaesthesia (ketamine 0.1 μg/g). All animals were individually identified by a passive integrated transponder. The animals were examined twice a week until neurological signs appeared, after which they were examined daily. Diseased animals were culled with carbon dioxide at the terminal stage of the disease, but before neurological impairment was such as to compromise their welfare, in particular their ability to drink and feed adequately. Survival time was calculated as the interval between inoculation and culling or death. After sacrifice, the brain from each animal was removed and cut sagittally into two parts: one stored at −80°C and one fixed in formalin.

Histological assessment was performed on formalin-fixed tissues as previously described [36]. Briefly, brains were trimmed at standard coronal levels, embedded in paraffin wax, cut at 6 μm and stained with haematoxylin and eosin. Vacuolar changes were scored in nine grey-matter areas of the brain on haematoxylin and eosin-stained sections, as previously described [36]. Vacuolation scores are derived from at least six individual voles per group and are reported as means ± standard error of the mean.

## Supporting Information

S1 Fig Lack of PrP^Sc^ amplification in negative controls seeded with healthy sheep brain homogenates. Three brain homogenates from 3 healthy sheep (indicated as A, B and C at the top of the blot) were serially 10-fold diluted and the last 3 dilutions (10^-5^, 10^-6^ and 10^-7^) were used as seeds in serial PMCA reactions using vole brain homogenate substrate. Products from the 7^th^ round were digested with PK and analyzed by Western blot with antibody SAF84. Bank vole PrP^C^ from (first lane of the blot) was loaded as a control.
(TIF)

S2 Fig Biochemical characterization of 18K and 14K PrP^Sc^. **A)** Epitope mapping of PrP^res^ from vole-adapted scrapie (BvScr), 18K and 14K/2. PrP^C^ from a negative vole brain homogenate not treated with PK was also included as control. Membranes probed with 9A2 and SAF32 antibodies are shown, as indicated on each blot. **B)** Deglycosylation of 18K and 14K/2 PrP^res^. Samples were PK digested and then subjected to deglycosylation as indicated at the top of the blot. Deglycosylated PrP^res^ was detected with mAb SAF84.
(TIF)
