# Supplementary figures and images for: Isolation of a Defective Prion Mutant from Natural Scrapie
Source: PLoS Pathog. 2016 Nov 23;12(11):e1006016. doi: 10.1371/journal.ppat.1006016 (PMC5120856; doi:10.1371/journal.ppat.1006016)

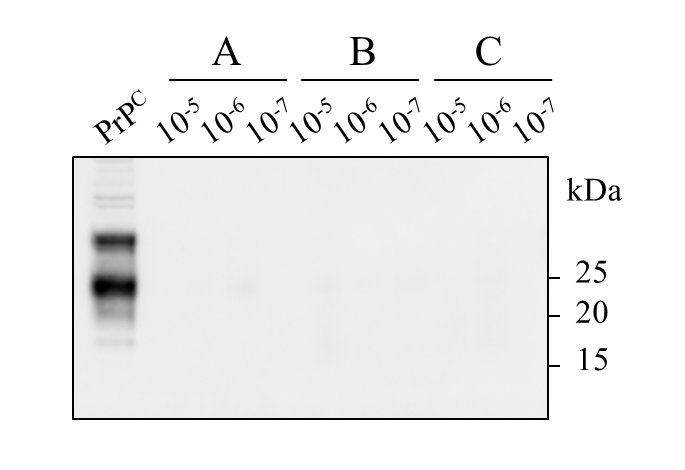

Supplement: S1 Fig — Three brain homogenates from 3 healthy sheep (indicated as A, B and C at the top of the blot) were serially 10-fold diluted and the last 3 dilutions (10-5, 10-6 and 10-7) were used as seeds in serial PMCA reactions using vole brain homogenate substrate. Products from the 7th round were digested with PK and analyzed by Western blot with antibody SAF84. Bank vole PrPC from (first lane of the blot) was loaded as a control. (TIF) [file ppat.1006016.s001.tif]

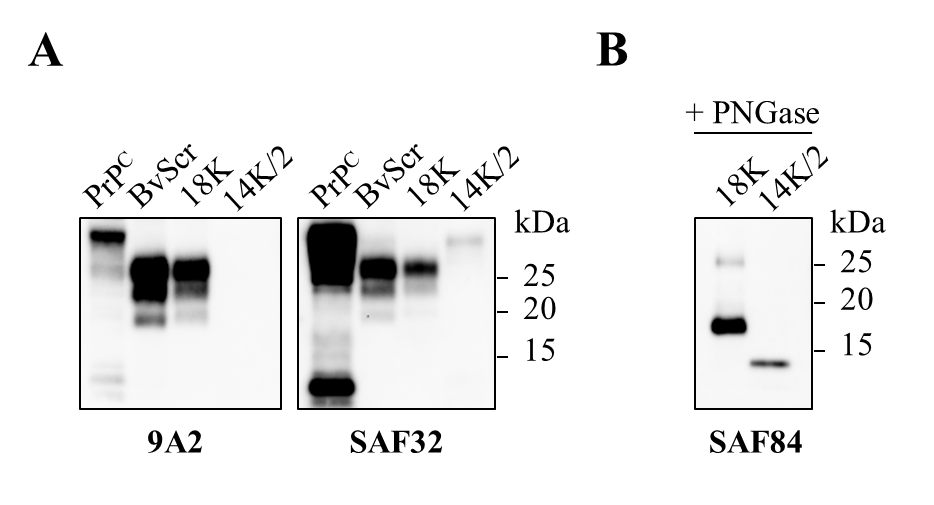

Supplement: S2 Fig — A) Epitope mapping of PrPres from vole-adapted scrapie (BvScr), 18K and 14K/2. PrPC from a negative vole brain homogenate not treated with PK was also included as control. Membranes probed with 9A2 and SAF32 antibodies are shown, as indicated on each blot. B) Deglycosylation of 18K and 14K/2 PrPres. Samples were PK digested and then subjected to deglycosylation as indicated at the top of the blot. Deglycosylated PrPres was detected with mAb SAF84. (TIF) [file ppat.1006016.s002.tif]
